# Newer Therapeutics to Selectively Kill *Clostridioides difficile* and Restore the Microbiome

**DOI:** 10.3390/idr18020034

**Published:** 2026-04-11

**Authors:** Guido Granata, Nicola Petrosillo

**Affiliations:** 1 Systemic and Immune Depression-Associated Infection Unit, National Institute for Infectious Diseases “L. Spallanzani”, I.R.C.C.S., 00149 Roma, Italy; 2 Infectious Disease Service, Fondazione Policlinico Universitario Campus Bio-Medico, 00127 Rome, Italy; n.petrosillo@policlinicocampus.it

**Keywords:** *Clostridioides difficile* infection management, prevention of recurrence of *Clostridioides difficile* infection, ibezapolstat, clinical trials, long term patients’ outcome, quality of life, live-JSLM, Rebyota, microbiome-based therapies, faecal microbiota transplantation, live biotherapeutic product, novel antimicrobials, human gut microbiota, bile acids

## Abstract

Background: The antibiotic ibezapolstat and the live biotherapeutic product live-JSLM are promising future approaches for treating *Clostridioides difficile* infection. Ibezapostat is a highly specific antibiotic for *Clostridioides difficile*, with minimal impact on the intestinal flora. Live-JSLM is designed to restore healthy intestinal microbiota, thus preventing recurrence of *Clostridioides difficile* infection. In this narrative review, we reviewed available data on ibezapostat and live-JSLM, considering that they are prototypes of two distinct, unique mechanisms of action against *Clostridioides difficile*. Methods: Data sources: PubMed and SCOPUS databases were searched from 1 January 2012 to 15 November 2025. Original articles reporting data on ibezapolstat and live-JSLM were included. Results: 31 studies were included. When compared to conventional anti-*Clostridioides difficile* antibiotics, ibezapolstat had a similar level of effectiveness and minimal impact on the gut microbiota. The available data confirm live-JSLM safety and efficacy in restoring the gut microbiota following the conclusion of the standard anti-*Clostridioides difficile* antibiotic regimen. Conclusions: The results on ibezapolstat efficacy are promising, but require confirmation in larger patient populations through double-blind, randomised phase III trials. In the near future, an integrated approach may enhance the management of *Clostridioides difficile* infection: starting with highly specific antibiotics, i.e., ibezapolstat, followed by microbiome-based therapies such as live-JSLM.

## 1. Introduction

*Clostridioides difficile* (*C. difficile*) is a Gram-positive, anaerobic, spore-forming bacterium, responsible for 12% of all healthcare-associated infections and recurrence rates reaching approximately 25% after an initial episode, escalating to 40% following the first recurrence [1].

Currently, *Clostridioides difficile* infection (CDI) represents a substantial burden worldwide, with over 400,000 infections and 200,000 hospitalizations annually in the United States [2] and around 124,000 cases and 3700 deaths annually in Europe [3].

Despite the considerable evolution of the therapeutic landscape for CDI in recent years, the management of CDI continues to represent a challenge in daily clinical practice [4]. The antibiotics vancomycin and fidaxomicin remain the recommended first-line options, but there are limitations to their effectiveness in preventing recurrence of CDI (rCDI), especially with vancomycin [5]. Fidaxomicin has demonstrated superiority over vancomycin in reducing rates of CDI recurrence [6]. Clinical guidelines increasingly recommend fidaxomicin as the preferred agent for initial or recurrent CDI [5]. Moreover, although fidaxomicin has a higher initial cost in comparison to vancomycin, its use can lead to fewer readmissions and lower overall healthcare costs due to reduced recurrence [5,6].

The high rate of rCDI with the currently available treatments has been demonstrated to have a detrimental effect on patients’ quality of life, to cause additional costs, and to increase overall mortality [1,2,3,4,5]. Furthermore, the occurrence of rCDI can lead to an increased risk of further rCDI episodes, often resulting in a deterioration of frailty in CDI patients experiencing a spiral of disease [1,2,3,4,5].

The occurrence of rCDI is driven by the disruption of the gut microbiota [7,8]. This disruption can be induced by broad-spectrum antibiotic treatment, leading to a loss of bacterial diversity and a decrease in the proportion of bacteria that are essential for bile acid metabolism [7,8]. These changes may result in elevated primary bile acid levels, facilitating *C. difficile* spore germination and onset of rCDI [7,8,9]. The disruption of microbiota is characterised by a weakening of antagonistic relationships between microbes and impaired metabolic functions, including a decrease in the production of secondary bile acids [7,8]. Secondary acids play a crucial role in the inhibition of the germination and growth of *C. difficile* spores, and therefore their decline can promote both CDI and rCDI [7,8]. To note, in CDI patients, the reconstitution of a balanced, species-rich gut microbiota has been demonstrated to reduce the likelihood of rCDI [7,8].

The faecal microbiota transplantation has been demonstrated to be effective in restoring gut microbiota and treating rCDI [9]. However, the implementation of faecal microbiota transplantation is complicated by logistical issues, including donor screening, stool processing, and patient monitoring for adverse effects. Additionally, the lack of standardisation across centres further complicates the process [9].

Recently, two novel therapies have been developed, ibezapostat and live-JSLM, having different, innovative mechanisms of action that can be considered novel therapeutic paradigms in the treatment and prevention of CDI.

Firstly, ibezapolstat is a new-generation antibiotic belonging to an entirely novel class. It is the first inhibitor of DNA polymerase IIIC, an enzyme that is crucial for the replication of *C. difficile*. Ibezapolstat is highly selective against *C. difficile* while preserving the rest of the gut microbiota. Ibezapolstat therefore represents a new generation of targeted, microbiota-sparing antibiotics.

Conversely, faecal microbiota live-JSLM is the first FDA approved live biotherapeutic product for preventing CDI recurrence [10]. This therapy involves a standardised suspension of faecal microbiota, designed to restore microbial biodiversity and correct the dysbiosis responsible for CDI recurrence. Live-JSLM can therefore be considered the prototype of new approaches based on the direct modulation of the microbiome.

This narrative review summarises the available data on the antibiotic ibezapolstat and the live biotherapeutic product live-JSLM to treat CDI and prevent rCDI.

## 2. Materials and Methods

The MEDLINE and SCOPUS databases were used to search for published articles on ibezapolstat and live-JSLM from 1 January 2012 to 15 November 2025. The search terms [(ibezapolstat)] and [(Rebyota) AND (live-jslm) AND (RBX2660)] were used in the MEDLINE database in separate searches. The SCOPUS database was searched using the following terms—ibezapolstat, Rebyota, live-jslm, and RBX2660—in multiple searches. No attempt was made to obtain information from unpublished studies.

The search strategy was intended to provide a structured overview of the literature rather than a systematic review process.

Quantitative and qualitative information was summarised by means of textual descriptions.

## 3. Results

In total, 31 studies were included in this review [11,12,13,14,15,16,17,18,19,20,21,22,23,24,25,26,27,28,29,30,31,32,33,34,35,36,37,38,39,40,41]. Supplementary Figure S1 shows the selection process of the included studies. Of the 31 included studies, 11 provided data on ibezapolstat [11,12,13,14,15,16,17,18,19,20,21] and 20 studies evaluated live-JSLM [22,23,24,25,26,27,28,29,30,31,32,33,34,35,36,37,38,39,40,41]. A summary description of the included studies is reported in Supplementary Tables S1 and S2.

### 3.1. Ibezapolstat

Ibezapolstat is a novel antibiotic and dichlorobenzyl purine analogue with a unique mechanism of action. Ibezapolstat binds to and inhibits the deoxyribonucleic acid (DNA) polymerase IIIC component of aerobic, Gram-positive bacteria, including *C. difficile* [11].

DNA polymerase IIIC is essential for replicative DNA synthesis in Gram-positive bacteria of the phylum Firmicutes, with a low guanine and cytosine content (i.e., fewer guanine and cytosine bases than adenine and thymine bases). Ibezapolstat binds to bacterial DNA polymerase III via an enzyme-specific aryl domain. Binding of the compound to this domain results in the formation of an inactive ternary complex comprising the inhibitor, DNA, and DNA polymerase IIIC. DNA polymerase IIIC is absent from the genomes of Gram-negative bacteria, making ibezapolstat a target with a restricted phylogeny and a narrow spectrum of activity [11].

Ibezapolstat spares common, low guanine and cytosine content gut commensal bacteria such as *Lachnospiraceae*, *Oscillospiraceae*, and *Coprobacillaceae* [12]. An in vitro study suggests that these bacteria are spared due to the phylogenetic variation in their DNA polymerase IIIC binding pocket residues [12].

A microbiological study conducted on a large collection of bacterial isolates confirmed ibezapolstat activity against *C. difficile* [13] and a study collecting 313 isolates from both in-hospital and community CDI patients reported ibezapolstat MIC^50^ and MIC^90^ of 4 mg/L [14].

Moreover, several studies confirmed that ibezapolstat exhibits high in vitro activity against *C. difficile* isolates belonging to various serotypes, with an MIC^50^ of 4 mg/L and an MIC^90^ ranging from 4 to 8 mg/L [11,15,16].

Recently, an animal model study compared the changes in the microbiome caused by ibezapolstat with other traditional anti-CDI antibiotics [17]. The changes in alpha and beta diversities after treatment with fidaxomicin and ibezapolstat were less pronounced than those observed with vancomycin or metronidazole [17].

A phase I trial assessed the pharmacokinetic profile of ibezapolstat in humans, with low systemic absorption and high concentrations in the colon, making ibezapolostat pharmacokinetics optimal for treating CDI [18].

A subsequent study was performed in order to assess the changes in the microbiome and bile acid changes associated with the administration of ibezapolstat, in comparison to those associated with vancomycin [19]. The faecal samples were obtained from the phase I healthy volunteer study [18]. Utilising metagenomic sequencing and mass spectrometry, the study found that both ibezapolstat and vancomycin exerted an effect on the human microbiome, but in distinct manners. A rapid increase in alpha diversity in the faecal microbiome was noted after starting ibezapolstat therapy, which was maintained after completion of therapy. After starting ibezapolstat, a decrease in the *Bacteroidetes* phylum was observed, with a concomitantly increased proportion of the *Firmicutes* phylum. Compared with the baseline, total primary bile acids decreased by a mean of 40.1 ng/mg of stool during ibezapolstat therapy (*p* < 0.001) [19].

A single-arm, open-label phase II trial was conducted to assess the clinical cure rates of CDI two days after the conclusion of treatment with ibezapolstat, as well as ibezapolstat safety [20]. The trial enrolled 10 adult CDI patients who received 450 mg of ibezapolstat orally at 12 h intervals for a period of 10 days. The patients were then observed for a 28-day follow-up period to assess the clinical response, identify any adverse events, and ascertain the status of the faecal microbiome. The initial clinical cure rate was reported in 10 out of 10 patients (100%). The mean time to resolution of diarrhoea was 5 days (range, 3–7 days). The sustained cure rate at 28 days was also 100% [20].

Recently, a larger phase II, randomised, double-blind, active-controlled trial compared ibezapolstat to vancomycin as a treatment for CDI patients [21]. The objective of the trial was to evaluate the efficacy, safety, pharmacokinetics, and associated microbiome changes in ibezapolstat in comparison with vancomycin for the treatment of adult CDI patients [21].

In this trial, 32 adult CDI patients were randomly assigned (1:1) to receive either ibezapolstat, 450 mg orally twice daily for 10 days, or vancomycin, 125 mg orally four times daily for 10 days. Participants were assessed for clinical response to treatment 2 days after the end of therapy and at a follow-up study visit to assess for sustained clinical cure at day 38. A subset of participants who volunteered for long-term follow-up were also reevaluated at days 56 and 84 [21]. Overall, 16 patients in the ibezapolstat arm and 14 CDI patients in the vancomycin arm were included in the efficacy evaluation. In total, 15 (94%) of the 16 participants in the ibezapolstat group had initial clinical cure, compared with 14 (100%) of 14 participants in the vancomycin group (treatment difference: −6.3%, 95% confidence interval: −30.7 to 19.4, *p*: 1.0). No participants in the ibezapolstat group had a recurrence assessed on day 28 after the end of treatment compared with 14% in the vancomycin group [21]. Moreover, 15 (94%) participants in the ibezapolstat group had sustained clinical cure compared with 12 (86%) in the vancomycin group (treatment difference: 8.0%, 95% confidence interval: −19.4 to 38.0, *p*: 0.59). Regarding safety analysis, both treatments were well tolerated with no drug-related serious adverse events. In total, 18% (3/17 participants) in the ibezapolstat group had mild adverse events, including gastro-oesophageal reflux disease and nausea. Bile acid analysis showed that participants in the vancomycin group had decreased primary bile acid conversion, leading to decreased ratios of secondary to primary bile acids, a shift not observed in the ibezapolstat group [21].

### 3.2. Live-JSLM

Faecal microbiota, live-JSLM, previously designated as RBX2660, is a single-dose, live biotherapeutic product comprising a consortium of microbes prepared from human faeces [10]. Live-JSLM is constituted of a liquid suspension, comprising a diverse set of microorganisms and is administered via rectal administration, with the objective of reducing the recurrence rate of CDI in adult CDI patients [10].

The phase II, double-blind, randomised, placebo-controlled PUNCH CD2 trial enrolled and randomised 127 patients with recurrent CDI, demonstrating efficacy in restoring the composition of the gut microbiome and reducing the risk of recurrent CDI [22,23].

A substudy of this trial was conducted to characterise the faecal bacterial microbiome before and after the live-JSLM administration [24,25]. Live-JSLM was found to be more effective than the placebo at restoring participant microbiomes [24,25].

Subsequent to this trial, open-label phase II clinical trials were performed [26]. The open-label trials comprised a total of 149 participants who were treated with live-JSLM, in comparison to a historical control group of patients. The trial comprised patients who had experienced two or more rCDI. The participants were administered two doses of live-JSLM rectally, with the doses administered seven days apart. The trial demonstrated that 78.9% (112/142) of patients administered with live-JSLM experienced no further instances of recurrent CDI for a period of 8 weeks following the conclusion of the study treatment, in contrast to the 30.7% (23/75) observed in the historical control group (*p* < 0.0001) [26].

Theoretically, exposure to broad-spectrum antibiotics after live-JSLM administration may diminish its beneficial effects. A post hoc analysis was performed on a subgroup of patients from the PUNCH open-label study who subsequently received non-CDI antibiotics after live-JSLM administration [27]. This subgroup comprised 43 patients who primarily received fluoroquinolones, cephalosporins, or penicillins to treat urinary or respiratory tract infections. The results were reassuring, with treatment response rates at eight weeks and one year higher than 90% [27]. A different post hoc analysis on a subgroup of 29 patients from the PUNCH CD2 trial confirmed the safety and efficacy of live-JSLM in rCDI and observed that live-JSLM reduces the abundance of antibiotic-resistant *Enterobacteriaceae* at 2 months after administration [28].

Subsequently, the PUNCH CD3 trial was conducted to demonstrate the effectiveness and safety of live-JSLM in treating patients with recurrent CDI. It was a large, prospective, randomised, double-blind, placebo-controlled, phase III study [29]. The participants were adults with rCDI who had completed one or more rounds of standard antibiotic therapy or had experienced two or more severe episodes of CDI resulting in hospitalisation within the past year. 267 patients were randomised 2:1 to receive live-JSLM or a placebo rectally, after completing a full course of antibiotic treatment for rCDI. Treatment success was defined as the absence of diarrhoea within 8 weeks of the treatment. Live-JSLM had a higher treatment success rate than placebo (70.6% versus 57.5%) and was well tolerated [29].

Post hoc analysis of the PUNCH CD3 trial demonstrated significant improvements in the quality of life of patients with a first recurrence of CDI who received live-JSLM [30,31].

Following the results of the PUNCH CD3 trial, the US Food and Drug Administration approved live-JSLM for the prevention of recurrent CDI in adults following standard anti-CDI antibiotic treatment.

Afterwards, a retrospective study was conducted, with the main aim to confirm the safety of live-JSLM in CDI patients [32]. Of 94 patients who were treated with live-JSLM, 82.8% responded at 8 weeks, and 88.7% of these patients had a sustained response at 6 months [32].

The PUNCH CD3-OLS was an open-label trial designed to assess the safety and efficacy of live-JSLM for the prevention of rCDI in a large real-world, clinical practice population [33].

A total of 793 participants were enrolled in the study, 697 of whom received live-JSLM [33]. At 8 weeks from the live-JSLM administration, 47.3% of the study participants had experienced mild or moderate gastrointestinal adverse events. Serious adverse events were reported by 3.9% of participants. In the PUNCH CD3-OLS study, live-JSLM achieved a sustained clinical response in 91% of participants at 6 months [33].

A post hoc subgroup analysis of the PUNCH CD3-OLS trial indicated that live-JSLM is also safe and effective as adjunctive treatment to prevent rCDI in patients with inflammatory bowel disease [34]. A post hoc analysis confirmed the safety and efficacy of live-JSLM in moderately immunocompromised CDI patients, including those with neoplasms, renal failure, HIV infection, and those assuming concomitant immunosuppressants, either glucocorticoids or selective immunosuppressants [35].

In the five clinical trials conducted to evaluate live-JSLM, it was found to be safe in rCDI patients [36,37].

Moreover, two studies were conducted to further assess the safety and clinical effectiveness of live-JSLM when administered via colonoscopy [38,39]. The CDI-SCOPE trial (NCT05831189) was a single-arm study that evaluated the administration of live-JSLM via colonoscopy [39]. The trial comprised 41 rCDI adult patients. The analysis revealed that 39 participants (95.1%) experienced treatment success. A total of five treatment-emergent adverse events were reported in 4/41 participants (9.8%), all of which were of a gastrointestinal nature and mild in severity [39].

The results of the studies that evaluated the microbiome changes in patients enrolled in the PUNCH trials demonstrated that the alterations in the microbiome of patients receiving live-JSLM were associated with a clinical response [40,41]. The patients who exhibited a positive response to the administration of live-JSLM had a higher proportion of *Bacteroidia* and *Clostridia* bacteria, accompanied by an escalation in alpha diversity [40,41]. Concurrently, a transition from primary to secondary bile acid dominance was reported [40,41].

## 4. Discussion

In an ideal scenario, a new innovative pharmaceutical compound to treat CDI should demonstrate comparable levels of activity against *C. difficile* to traditional anti-CDI antibiotics, yet not exhibit any activity against the essential gut microbiota, thereby preventing gut dysbiosis. As an alternative approach, the ideal compound should rapidly and safely restore a healthy microbiota following the conclusion of the standard anti-CDI antibiotic regimen, thereby halting the germination of *C. difficile* and preventing rCDI.

Thus, consider that live-JSLM and ibezapolstat represent two innovative, promising, and complementary approaches with different models of action:

(1) Restoration of the microbiota through a standardised live biotherapeutic product, i.e., live-JSLM.
(2) Selective and targeted inhibition of bacterial replication via a new molecular target, not shared by currently used antibiotics, i.e., ibezapolstat.

Overall, the current evidence regarding the novel antibiotic ibezapolstat appears encouraging. Ibezapolstat was reported to be active against *Clostridioides difficile* with a low impact on the beneficial gut microbiota [11,15,16,17,20].

Notably, the observed reduction in primary bile acids, along with an improved secondary-to-primary bile acid ratio, suggests that ibezapolstat administration may lower the risk of CDI recurrence [7,20]. Although the results on ibezapolstat efficacy are promising, they require confirmation in larger patient populations through double-blind, randomised phase III trials.

As for live-JSLM, the available data suggest it may help in restoring a healthy microbiota following the conclusion of the standard anti-CDI antibiotic regimen, thereby halting the germination of *C. difficile* and preventing rCDI [33,34,35]. Importantly, clinical trials assessing live-JSLM’s safety have consistently confirmed its favourable safety profile [36]. The administration of live-JSLM rectally or via colonoscopy is practical, safe, and effective [33,39].

Among the limitations of our narrative review, the included studies were highly diverse and had different objectives. Furthermore, some included studies had a small patient population. Moreover, a major limitation is that we did not perform a validated risk-of-bias evaluation with a specific tool for each study. The included studies are highly heterogeneous, including a wide range of study types—from in vitro studies and animal models to early-phase and randomised clinical trials. This mixed evidence base and the different levels of evidence present significant limitations regarding the interpretation of findings.

Acknowledging these pitfalls, the current evidence concerning the novel antibiotic ibezapolstat and the live biotherapeutic agent live-JSLM is promising.

In our opinion, in the near future, an integrated approach may enhance the management of CDI and better address the underlying pathophysiology of microbiome dysbiosis, preventing CDI recurrence [9]. A hypothetical future approach could involve two steps: first, starting with anti-*C. difficile* antibiotics, i.e., ibezapolstat, followed by microbiome-based therapies such as live-JSLM [9].

## Data Availability

The original data presented in the study are openly available online in PubMed, Embase, Scopus, and Cochrane Library.

## Supplementary Materials

The following supporting information can be downloaded at: https://www.mdpi.com/article/10.3390/idr18020034/s1, Figure S1: Flowchart depicting the selection process of studies included in the review. Table S1: Description of the included studies on ibezapolstat. Table S2: Description of the included studies on live-JSLM.

## References

[1] Magill S.S., Edwards J.R., Bamberg W., Beldavs Z.G., Dumyati G., Kainer M.A., Lynfield R., Maloney M., McAllister-Hollod L., Nadle J. (2014). Emerging Infections Program Healthcare-Associated Infections and Antimicrobial Use Prevalence Survey Team. Multistate point-prevalence survey of health care-associated infections. N. Engl. J. Med..

[2] Guh A.Y., Mu Y., Winston L.G., Johnston H., Olson D., Farley M.M., Wilson L.E., Holzbauer S.M., Phipps E.C., Dumyati G.K. (2020). Emerging Infections Program *Clostridioides difficile* Infection Working Group. Trends in U.S. burden of *Clostridioides difficile* infection and outcomes. N. Engl. J. Med..

[3] Strategies and Guidelines for *Clostridioides difficile* Infections. http://www.ecdc.europa.eu/en/clostridium-difficile-infections/facts.

[4] Smits W.K., Garey K.W., Riley T.V., Han Y.W., Young V.B., Heritage J., Lillis C.J., Lyras D., Rupnik M., Johnson S. (2025). *Clostridioides difficile* should be considered a bacterial priority pathogen. Lancet Microbe.

[5] Di Bella S., Sanson G., Monticelli J., Zerbato V., Principe L., Giuffrè M., Pipitone G., Luzzati R. (2024). *Clostridioides difficile* infection: History, epidemiology, risk factors, prevention, clinical manifestations, treatment, and future options. Clin. Microbiol. Rev..

[6] Bednárik D.S., Földvári-Nagy K.C., Simon V., Rancz A., Gede N., Veres D.S., Paraskevopoulos P., Schnabel T., Erőss B., Hegyi P. (2025). Comparative effectiveness of different therapies for *Clostridioides difficile* infection in adults: A systematic review and network meta-analysis of randomized controlled trials. Lancet Reg. Health Eur..

[7] Buffie C.G., Pamer E.G. (2013). Microbiota-mediated colonization resistance against intestinal pathogens. Nat. Rev. Immunol..

[8] Collins S.L., Stine J.G., Bisanz J.E., Okafor C.D., Patterson A.D. (2023). Bile acids and the gut microbiota: Metabolic interactions and impacts on disease. Nat. Rev. Microbiol..

[9] Granata G., Petrosillo N. (2024). Tackling *Clostridioides difficile* (CD): Current evidences and future directions in the treatment of CD infections. Lancet Reg. Health Eur..

[10] REBYOTA (Bacrodiome-Mcrb) (2022). Prescribing Information.

[11] Murray B., Wolfe C., Marra A., Pillar C., Shinabarger D. (2020). In vitro activity of the novel antibacterial agent ibezapolstat (ACX-362E) against *Clostridioides difficile*. J. Antimicrob. Chemother..

[12] McPherson J.K., Hurdle J.G., Baker M.L., Hussain T., Kumar A., Garey K.W. (2025). The microbiome-restorative potential of ibezapolstat for the treatment of *Clostridioides difficile* infection is predicted through variant PolC-type DNA polymerase III in *Lachnospiraceae* and *Oscillospiraceae*. Antimicrob. Agents Chemother..

[13] van Eijk E., Boekhoud I.M., Kuijper E.J., Bos-Sanders I.M.J.G., Wright G., Smits W.K. (2019). Genome location dictates the transcriptional response to PolC inhibition in *Clostridium difficile*. Antimicrob. Agents Chemother..

[14] Schwartz O., Azrad M., Peretz A. (2025). In vitro susceptibility of clinical *Clostridioides difficile* isolates in Israel to metronidazole, vancomycin, fidaxomicin, ridinilazole and ibezapolstat. BMC Gastroenterol..

[15] Dvoskin S., Xu W.-C., Brown N.C., Yanachkov I.B., Yanachkova M., Wright G.E. (2012). A novel agent effective against *Clostridium difficile* infection. Antimicrob. Agents Chemother..

[16] Bassères E., Eubank T.A., Begum K., Alam M.J., Jo J., Le T.M., Lancaster C.K., Gonzales-Luna A.J., Garey K.W. (2024). Antibacterial activity of ibezapolstat against antimicrobial-resistant clinical strains of *Clostridioides difficile*. Antimicrob. Agents Chemother..

[17] Wolfe T.M., Jo J., Pinkham N.V., Garey K.W., Walk S.T. (2025). The impact of ibezapolstat and other *Clostridioides difficile* infection-relevant antibiotics on the microbiome of humanized mice. Antimicrob. Agents Chemother..

[18] Garey K.W., Begum K., Lancaster C., Gonzales-Luna A., Bui D., Mercier J., Yue C.S., Ducharme M.P., Hu M., Vince B. (2020). A randomized, double-blind, placebo-controlled, single and multiple ascending dose Phase 1 study to determine the safety, pharmacokinetics and food and faecal microbiome effects of ibezapolstat administered orally to healthy subjects. J. Antimicrob. Chemother..

[19] McPherson J., Hu C., Begum K., Wang W., Lancaster C., Gonzales-Luna A.J., Loveall C., Silverman M.H., Alam M.J., Garey K.W. (2022). Functional and metagenomic evaluation of ibezapolstat for early evaluation of anti-recurrence effects in *Clostridioides difficile* infection. Antimicrob. Agents Chemother..

[20] Garey K.W., McPherson J., Dinh A.Q., Hu C., Jo J., Wang W., Lancaster C.K., Gonzales-Luna A.J., Loveall C., Begum K. (2022). Efficacy, safety, pharmacokinetics, and microbiome changes of ibezapolstat in adults with *Clostridioides difficile* infection: A Phase 2a multicenter clinical trial. Clin. Infect. Dis..

[21] Eubank T.A., Jo J., Alam M.J., Begum K., McPherson J.K., Le T.M., Horvath T.D., Haidacher S.J., Poggio E.C., Lin R. (2025). Ibezapolstat Phase 2 Investigator Group. Efficacy, safety, pharmacokinetics, and associated microbiome changes of ibezapolstat compared with vancomycin in adults with *Clostridioides difficile* infection: A Phase 2b, randomised, double-blind, active-controlled, multicentre study. Lancet Microbe.

[22] Dubberke E.R., Lee C.H., Orenstein R., Khanna S., Hecht G., Gerding D.N. (2018). Results from a randomized, placebo-controlled clinical trial of a RBX2660—A microbiota-based drug for the prevention of recurrent *Clostridium difficile* infection. Clin. Infect. Dis..

[23] Dubberke E.R., Orenstein R., Khanna S., Guthmueller B., Lee C. (2023). Final results from a Phase 2b randomized, placebo-controlled clinical trial of RBX2660: A microbiota-based drug for the prevention of recurrent *Clostridioides difficile* infection. Infect. Dis. Ther..

[24] Blount K.F., Shannon W.D., Deych E., Jones C. (2019). Restoration of bacterial microbiome composition and diversity among treatment responders in a Phase 2 trial of RBX2660: An investigational microbiome restoration therapeutic. Open Forum Infect. Dis..

[25] Orenstein R., Dubberke E., Hardi R., Ray A., Mullane K., Pardi D.S., Ramesh M.S., Kelly C., Mariani P., Misra B. (2016). Safety and durability of RBX2660 (microbiota suspension) for recurrent *Clostridium difficile* infection: Results of the PUNCH CD study. Clin. Infect. Dis..

[26] Orenstein R., Dubberke E.R., Khanna S., Lee C.H., Yoho D., Johnson S., Hecht G., DuPont H.L., Gerding D.N., Blount K.F. (2022). Durable reduction of *Clostridioides difficile* infection recurrence and microbiome restoration after treatment with RBX2660: Results from an open-label Phase 2 clinical trial. BMC Infect. Dis..

[27] Reveles K.R., Gonzales-Luna A.J., Golan Y., Alonso C.D., Guthmueller B., Tan X., Bidell M.R., Pokhilko V., Crawford C.V., Skinner A.M. (2024). Efficacy of fecal microbiota, live-jslm (REBYOTA^®^), among patients exposed to non-*Clostridioides difficile* infection antibiotics: Post hoc subgroup analysis of a Phase 2 open-label study. Open Forum Infect. Dis..

[28] Langdon A., Schwartz D.J., Bulow C., Sun X., Hink T., Reske K.A., Jones C., Burnham C.-A.D., Dubberke E.R., Dantas G. (2021). CDC Prevention Epicenter Program. Microbiota restoration reduces antibiotic-resistant bacteria gut colonization in patients with recurrent *Clostridioides difficile* infection from the open-label PUNCH CD study. Genome Med..

[29] Khanna S., Assi M., Lee C., Yoho D., Louie T., Knapple W., Aguilar H., Garcia-Diaz J., Wang G.P., Berry S.M. (2022). Efficacy and safety of RBX2660 in PUNCH CD3, a Phase III, randomized, double-blind, placebo-controlled trial with a Bayesian primary analysis for the prevention of recurrent *Clostridioides difficile* infection. Drugs.

[30] Feuerstadt P., Allegretti J.R., Dubberke E.R., Guo A., Harvey A., Yang M., Garcia-Horton V., Fillbrunn M., Tillotson G., Bancke L.L. (2024). Efficacy and health-related quality of life impact of fecal microbiota, live-jslm: A post hoc analysis of PUNCH CD3 patients at first recurrence of *Clostridioides difficile* infection. Infect. Dis. Ther..

[31] Garey K.W., Dubberke E.R., Guo A., Harvey A., Yang M., García-Horton V., Fillbrunn M., Wang H., Tillotson G.S., Bancke L.L. (2023). Effect of fecal microbiota, live-jslm (REBYOTA^®^) on health-related quality of life in patients with recurrent *Clostridioides difficile* infection: Results from the PUNCH CD3 clinical trial. Open Forum Infect. Dis..

[32] Feuerstadt P., Harvey A., Yoho D.S., Garcia-Diaz J.B., Knapple W.L., Bancke L. (2023). Retrospective analysis of the safety and efficacy of fecal microbiota, live-jslm (REBYOTA™) administered under enforcement discretion to patients with *Clostridioides difficile* infection. Open Forum Infect. Dis..

[33] Feuerstadt P., Chopra T., Knapple W., Van Hise N.W., Dubberke E.R., Baggott B., Guthmueller B., Bancke L., Gamborg M., Steiner T.S. (2025). PUNCH CD3-OLS: A Phase 3 prospective observational cohort study to evaluate the safety and efficacy of fecal microbiota, live-jslm (REBYOTA) in adults with recurrent *Clostridioides difficile* infection. Clin. Infect. Dis..

[34] Allegretti J.R., Feuerstadt P., Knapple W.L., Orenstein R., Pinton P., Sheh A., Khanna S. (2025). Safety and efficacy of fecal microbiota, live-jslm (REBYOTA^®^), for the prevention of recurrent *Clostridioides difficile* infection in participants with inflammatory bowel disease in PUNCH CD3-OLS. Inflamm. Bowel Dis..

[35] Alonso C.D., Tillotson G.S., Bidell M.R., Guthmueller B., Hoeyer F., Fischer M., Dubberke E.R. (2025). Safety and efficacy of fecal microbiota, live-jslm, in preventing recurrent *Clostridioides difficile* infection in participants who were mildly to moderately immunocompromised in the Phase 3 PUNCH CD3-OLS study. Open Forum Infect. Dis..

[36] Lee C., Louie T., Bancke L., Guthmueller B., Harvey A., Feuerstadt P., Khanna S., Orenstein R., Dubberke E.R. (2023). Safety of fecal microbiota, live-jslm (REBYOTA™) in individuals with recurrent *Clostridioides difficile* infection: Data from five prospective clinical trials. Ther. Adv. Gastroenterol..

[37] Feuerstadt P., Crawford C.V., Tan X., Pokhilko V., Bancke L., Ng S., Guthmueller B.A., Bidell M.R., Tillotson G., Johnson S. (2024). Fecal microbiota, live-jslm for the prevention of recurrent *Clostridioides difficile* infection: Subgroup analysis of PUNCH CD2 and PUNCH CD3. J. Clin. Gastroenterol..

[38] Knapple W.L., Yoho D.S., Sheh A., Thul J., Feuerstadt P. (2024). Retrospective subgroup analysis of fecal microbiota, live-jslm (REBYOTA^®^) administered by colonoscopy under enforcement discretion for the prevention of recurrent *Clostridioides difficile* infection. Ther. Adv. Gastroenterol..

[39] Khanna S., Yoho D., Van Handel D., Clark B.J., Awad T., Guthmueller B., Armandi D., Knapple W., Safdar N., Baggott B. (2025). Safety and effectiveness of fecal microbiota, live-jslm (REBYOTA^®^) administered by colonoscopy for prevention of recurrent *Clostridioides difficile* infection: 8-week results from CDI-SCOPE, a single-arm, Phase IIIb trial. Ther. Adv. Gastroenterol..

[40] Papazyan R., Ferdyan N., Srinivasan K., Gonzalez C., Shannon W.D., Blount K., Fuchs B.C. (2023). Human fecal bile acid analysis after investigational microbiota-based live biotherapeutic delivery for recurrent *Clostridioides difficile* infection. Microorganisms.

[41] Blount K.F., Papazyan R., Ferdyan N., Srinivasan K., Gonzalez C., Shannon W.D., Fuchs B.C. (2025). Microbiome and metabolome restoration after administration of fecal microbiota, live-jslm (REBYOTA) for preventing recurrent *Clostridioides difficile* infection. J. Infect. Dis..

