# Supplementary material for: Newer Therapeutics to Selectively Kill Clostridioides difficile and Restore the Microbiome
Source: Infect Dis Rep. 2026 Apr 11;18(2):34. doi: 10.3390/idr18020034 (PMC13116874; doi:10.3390/idr18020034)
Supplement: Supplementary file 1 [file idr-18-00034-s001.zip › idr-4160431-supplementary.pdf]

infectious disease reports

MDPI

Supplementary Figure S1. Flowchart depicting the selection process of studies included in the review.

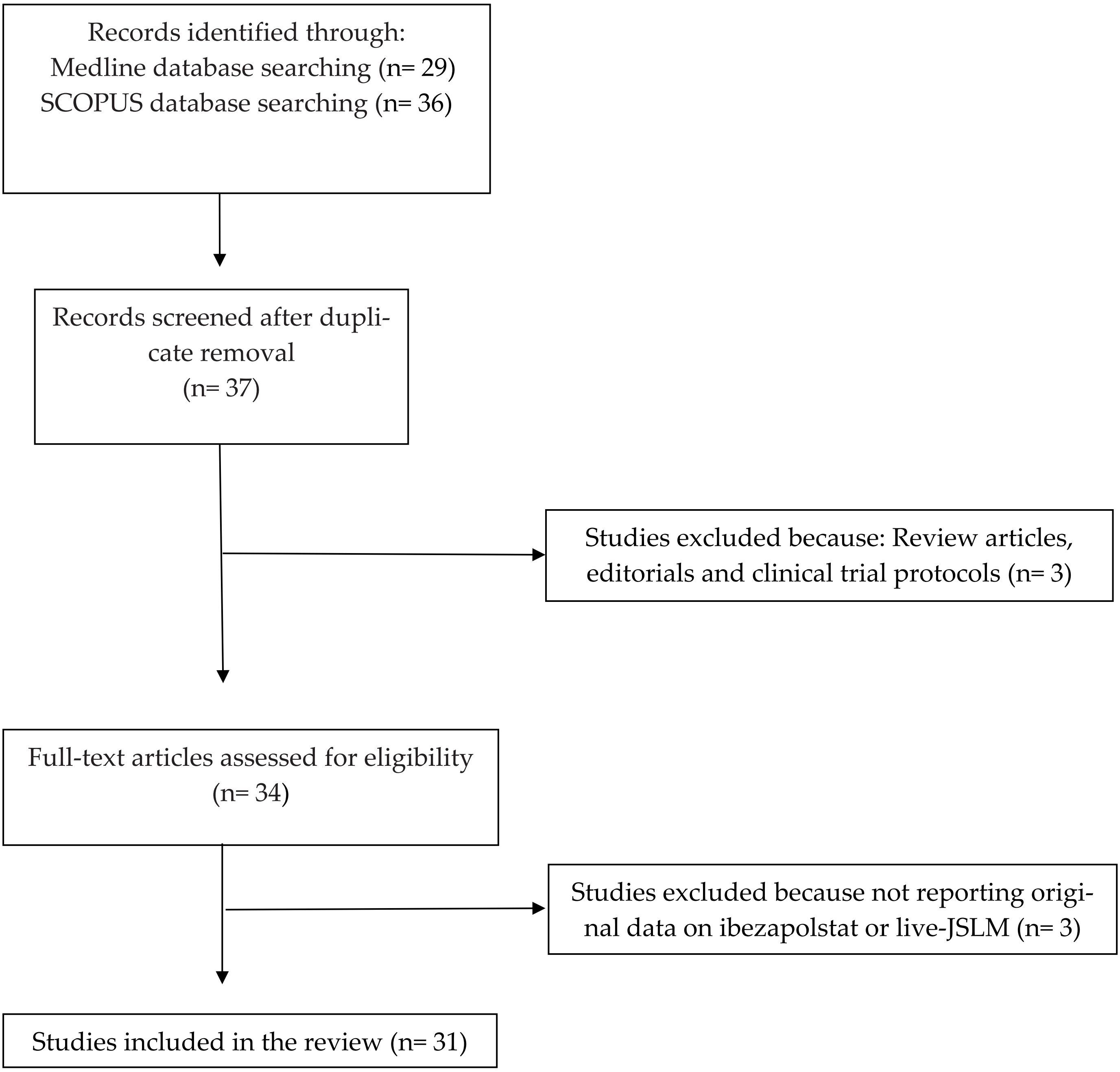

Supplementary Table S1. Description of the included studies on ibezapolstat.

Author, year and Country, Quality appraisal/ Overall risk of bias	Study Type	Setting, study period	Study Population, Age (Mean), Sex (% Male)	Study aim	Study design	Study results
Murray B et al. 2020. US, High reliability [11]	*In vitro* activity evaluation	Microbiology laboratory, US, 2020	104 isolates of *C. difficile*	To evaluate ibezapolstat in vitro activity against a panel of *C. difficile* isolates	The bactericidal activity of ibezapolstat was evaluated by determining the minimum bactericidal concentration. Time–kill kinetic assays were additionally performed with metronidazole and vancomycin as comparators	The overall $MIC_{50/90}$ for ibezapolstat against evaluated *C. difficile* was 2/4 mg/L, compared with 0.5/4 mg/L for metronidazole, 1/4 mg/L for vancomycin and 0.5/2 mg/L for fidaxomicin. Ibezapolstat demonstrated an MIC range of 1–8 mg/L, with an $MIC_{50/90}$ of 4/4 mg/L
McPherson JK et al. 2025. US High reliability [12]	*In silico* study	Microbiology laboratory, US 2025	-	To evaluate ibezapolstat spectrum of activity	Ibezapolstat susceptibility to human commensal microbiota was predicted using genomic analysis and polymerase IIIC	Amino acid phylogenetic tree identified certain residues that were phylogenetically variant in *Lachnospiraceae, Oscillospiraceae,* and *Erysipelotrichales* and conserved in *C. difficile*. Predicting ibezapolstat sparing of *Lachnospiraceae, Oscillospiraceae,* and *Coprobacillaceae*

					phylogenetic tree construction	
van Eijk E et al. 2019 the Netherlands High reliability [13]	*In vitro* activity evaluation	Microbiology laboratory in Leiden, 2025	363 *C. difficile* clinical isolates collected in the framework of a pan-European study	To assess the ibezapolstat efficacy against a large collection of clinical isolates	Antibiotic activity was evaluated by agar dilution method, RNA sequencing analyses	Ibezapolstat efficacy confirmed against a large collection of clinical isolates
Schwartz O et al. 2025 Israel Very high quality [14]	*In vitro* activity evaluation	Microbiology laboratory in Leiden, 2025	313 *C. difficile* isolates recovered from patients at both community and hospital medical centers across Israel	To assess the *in vitro* susceptibility of clinical isolates to two recently developed antibiotics, ridinilazole and ibezapolstat and to standard-of-care antibiotics	Isolates typed by multi-locus sequencing typing. Susceptibility to metronidazole and vancomycin was determined by Etest. Susceptibility to fidaxomicin, ridinilazole and ibezapolstat by agar dilution	Ibezapolstat had an $MIC_{50/90}$ of 4 mg/L
Dvoskin S et al. 2012. US [15]	Animal model study	Animal facility, US	60 golden Syrian hamsters	To evaluate ibezapolstat activity against CDI in the animal model	Ibezapolstat was administered by oral gavage to groups of 6 animals, starting 17 hours after *C. difficile* spore administration and continuing twice daily for 3 days	Ibezapolstat was effective in improving survival in the animal CDI model

Bassères E et al. 2024 US Very high reliability [16]	*In vitro* activity evaluation	Microbiology laboratory, US	100 clinical strains of *C. difficile*, of which 59 had reduced susceptibility to other antibiotics	To test the antibacterial properties of ibezapolstat against drug-resistant *C. difficile* strains	Agar dilution method and time-kill kinetic studies	The overall ibezapolstat MIC*50/90* values for evaluated C. difficile strains were 4/8 μg/mL. MIC50/90 values did not differ based on non-susceptibility to antibiotic class or number of classes to which strains were non-susceptible
Wolfe TM et al. 2025 US [17]	Animal model study	Animal facility, US	36 germ-free mice	To compare microbiome changes associated with ibezapolstat to other clinically used CDI antibiotics	Groups of germ-free mice received a fecal microbiota transplant from one of two healthy human donors and were subsequently exposed to either ibezapolstat, vancomycin, fidaxomicin, metronidazole, or no antibiotic (control). 16S rRNA encoding gene sequencing of temporally collected stool samples was used to compare the gut microbiome perturbations	In microbiome-humanized mice, ibezapolstat and fidaxomicin had smaller effects on gut microbiome diversity than vancomycin and metronidazole
Garey KW et al. 2020 US	Randomized, double-blind,	Clinical research unit in the US	62 subjects, aged 31 ± 7 years (45% female; average BMI: 25 ± 3 kg/m^2)	To assess the safety, tolerability and pharmacokinetics of	Subjects and investigators were blinded to ibezapolstat or placebo. The third part of the study was a	Ibezapolstat was well tolerated with a safety signal similar to placebo. Minimal systemic absorption with the majority of plasma concentrations less than 1 μg/mL. Ibezapolstat concentrations of

Overall risk of bias: Low [18]	placebo-controlled, single and multiple ascending dose Phase I study			ibezapolstat in healthy volunteers	multiday, ascending dose study up to 300 mg and 450 mg a day for 10 days	2000 µg/g of stool were observed by Day 2 and for the remainder of the dosing time period
McPherson J et al. 2022 US Overall risk of bias: Some concerns [19]	Phase I study subgroup analysis	Clinical research unit in the US	22 healthy volunteers, who were given either ibezapolstat or vancomycin.	To assess the changes in the microbiome and bile acid changes associated with the administration of ibezapolstat, in comparison to those associated with vancomycin	Stool samples were evaluated for microbiome changes and bile acid concentrations by functional and metagenomic analyses	Ibezapolstat 450 mg and vancomycin, but not ibezapolstat 300 mg, showed statistically significant changes in alpha diversity over time compared to that of a placebo. Vancomycin had a more wide-ranging effect on the microbiome, characterized by an increased proportion of *Gammaproteobacteria*. Ibezapolstat demonstrated an increased proportion of *Actinobacteria*, including the *Bifidobacteriaceae* family. Vancomycin was associated with significant increases in primary bile acids as well as primary/secondary bile acid ratios
Garey KW et al. 2022 US Overall risk of bias: Some	Single-arm, open-label, phase II study	4 US centers	10 adults CDI patients with a mean age of 49 years	To assess clinical cure rates and adverse events. Secondary objectives were to evaluate	Patients received ibezapolstat 450 mg orally every 12 hours for 10 days and followed for an additional 28	10 of 10 patients achieved sustained clinical cure. Plasma levels of ibezapolstat ranged from 233 to 578 ng/mL while mean (standard deviation) fecal levels were 416 (494) µg/g stool by treatment day 3 and >1000 µg/g stool by days 8-10.

concerns [20]				plasma/fecal pharmacokinetics, microbiologic eradication, microbiome and bile acid effects	days to assess study objectives	A rapid increase in alpha diversity in the fecal microbiome was noted after starting ibezapolstat therapy, which was maintained after completion of therapy. A decrease in *Bacteroidetes* phylum with a concomitantly increased proportion of Firmicutes phylum. Compared with baseline, total primary bile acids decreased by a mean of 40.1 ng/mg stool during therapy ($p< 0.001$)
Eubank TA et al. 2025 US Overall risk of bias: Low [21]	Phase II, randomised, double-blind, active-controlled trial	15 centres, primarily outpatient clinics and hospitals, in the US	32 adult CDI patients	To evaluate the efficacy, safety, pharmacokinetics, and associated microbiome changes of ibezapolstat in comparison with vancomycin for the treatment of adult patients with CDI	Patients were randomly assigned (1:1) to receive either ibezapolstat, 450 mg orally twice daily for 10 days, or vancomycin, 125 mg orally four times daily for 10 day. A subset of participants who volunteered for long-term follow-up were reevaluated at days 56 and 84	94% of the participants in the ibezapolstat group had initial clinical cure, compared with 100% in the vancomycin group (treatment difference: −6.3%, 95% confidence interval: −30.7 to 19.4, p: 1.0). No participants in the ibezapolstat group had a recurrence assessed on day 28 after the end of treatment compared with 14% in the vancomycin group. 94% of the participants in the ibezapolstat group had sustained clinical cure compared with 86% in the vancomycin group (treatment difference: 8.0%, 95% confidence interval: -19.4 to 38.0, p: 0.59). Regarding safety analysis, both treatments were well tolerated with no drug-related serious adverse events

CDI: *Clostridioides difficile* infection. MIC: Minimum inhibitory concentration.

Supplementary Table S2. Description of the included studies on live-JSLM.

Author, year and Country, Quality appraisal/ Overall risk of bias	Study Type	Setting, study period	Study Population, Age (Mean), Sex (% Male)	Study aim	Study design	Study results
Dubberke ER et al. 2018. US Overall risk of bias: Low [22]	Randomized, placebo-controlled phase II trial (NCT02299570)	21 centers in the US and Canada from December 2014 through November 2015	150 adults with 2 or more rCDI	To demonstrate the efficacy and safety of 1 or 2 doses of live-JSLM to prevent recurrent CDI among patients with a history of multiple rCDI	Patients were randomized at a 1:1:1 ratio to receive: 2 doses of live-JSLM; 2 doses of placebo or 1 dose of live-JSLM followed by 1 dose of placebo	Treatment success rates were 80% (4/5) and 78% (38/49) for participants who received 1 or 2 open-label live-JSLM treatments, respectively. The overall efficacy for live-JSLM -treated participants was 88.8%. Adverse events did not differ significantly among treatment groups
Dubberke ER et al. 2023. US Overall risk of bias: Low [23]	Prospective, multicenter, randomized, double-blinded, placebo-controlled, three-arm phase II clinical trial	21 centers in the US and Canada from December 2014 through November 2015	Recurrent CDI adult patients with at least 3 episodes of CDI and at least 2 rounds of standard antibiotic treatment or had at least 2 episodes of severe CDI resulting in	To evaluate the efficacy and safety of live-JSLM for the reduction of recurrent CDI compared to placebo	Patients were randomized 1:1:1 to receive: two doses of live-JSLM; 2 doses of placebo; or 1 dose of live-JSLM and one dose of placebo. Treatment success was prevention of recurrence, defined as absence of diarrhea and no re-treatment for CDI any time after the first dose until 8 weeks after the	Treatment success at 8 weeks, was 56.8% (25/45) of participants who received one dose of live-JSLM, 55.6% (25/45) of participants who received two doses of live-JSLM and 43.2% (19/44) of participants who received 2 doses of placebo. The safety profile of live-JSLM was similar to the placebo group

	(NCT02299570)		hospitalization		second dose of the study treatment	
Blount KF et al. 2019. US Overall risk of bias: Some concerns [24]	Subgroup analysis of the randomized, double-blinded placebo-controlled phase II PUNCH CD2 trial (NCT02299570)	PUNCH CD2 trial was performed across 21 centers in the US and Canada from December 2014 through November 2015	58 patients from the PUNCH CD2 trial	To characterize the fecal bacterial microbiome before and after treatment among live-JSLM - or placebo-treated responders in the PUNCH CD2 trial	Samples were sequenced using 16S methods, and the resulting relative abundance data were fit to a Dirichlet-multinomial distribution to determine group mean relative taxonomic abundance and overdispersion at the class level. Alpha diversity was determined for all samples	Prevention of recurrent CDI with live-JSLM was associated with restorative microbiome changes. Live-JSLM was more effective than placebo at restoring participant microbiomes. At study entry, subjects' microbiomes were dominated by *Gammaproteobacteria* and *Bacilli*, with low abundance of *Bacteroidia* and *Clostridia*. After treatment, *Bacteroidia*, *Clostridia*, and alpha diversity increased among live-JSLM responders, concomitant with a decrease of *Gammaproteobacteria* and *Bacilli*
Orenstein R et al. 2021 US Overall risk of bias: Some concerns [25]	Prospective open-label study – PUNCH CD study	11 US medical centers, between 15 August 2013 and 16 December 2013	40 adult patients with rCDI who had either at least 2 recurrences after a primary episode and had completed a least 2 courses of standard oral antibiotic therapy for CDI or had at least 2 episodes of	To assess the safety of live-JSLM at 6 months and its effectiveness at resolving multiply recurrent CDI at 8 weeks after the last live-JSLM administration	Patients having been on a variety of previously unsuccessful antimicrobial regimens prior to screening. Following completion of a 10- to 14-day course of oral antibiotics for CDI, the last 7 days of which were standardized to oral vancomycin 125 mg 4 times daily, followed by a 24- to 48-hour washout period, a single dose of live-JSLM was	Overall efficacy was 87.1% (16 with 1 dose and 11 with 2 doses). Of 188 reported adverse events, diarrhea, flatulence, abdominal pain or cramping, and constipation were most common. The frequency and severity of adverse events decreased over time

			severe CDI resulting in hospitalization		administered via enema. 60 days follow-up	
Orenstein R et al. 2022 US Overall risk of bias: Some concerns [26]	Phase II retrospective, open-label study PUNCH study (NCT02589847)	29 medical centers in the United States and Canada	149 adult patients with recurrent CDI	To evaluate the safety, efficacy, and durability of live-JSLM, in comparison to a closely-matched historical control cohort	Patients were administered two doses of live-JSLM rectally, with the doses administered 7 days apart. Patients were compared to a historical control group of patients	78.9% (112/142) of patients administered with live-JSLM experienced no further instances of recurrent CDI for a period of 8 weeks following the conclusion of the study treatment, in contrast to the 30.7% (23/75) observed in the historical control group ($p<0.0001$)
Reveles KR et al. 2024 US Overall risk of bias: Some concerns [27]	Post hoc analysis of the phase II PUNCH Open-Label study (NCT02589847)	29 medical centers in the United States and Canada	97 participants from the phase II PUNCH open-label study who subsequently received non-CDI antibiotics	To evaluate the long-term clinical response to live-JSLM	Participants in PUNCH Open-Label who received non-CDI antibiotics after live-JSLM administration were included in this subgroup analysis. Treatment response was defined as the absence of CDI diarrhea needing retreatment at the last evaluable time point (8 weeks, 6 months, 1 year, or 2 years) after live-JSLM administration	Live-JSLM was safe and efficacious in preventing rCDI as compared with a historical control group. 91% (88/97) of live-JSLM responders remained CDI recurrence-free 2 years after administration
Langdon A et al. 2021 US Overall risk of bias: Some concerns	Subgroup analysis from the phase II open-label clinical trial	11 centers in the US	Fecal specimens from 29 of the subjects included in the PUNCH CD study. Patients with	To evaluate the safety and efficacy of live-JSLM on rCDI	Fecal samples in this study were derived from a phase II prospective open-label cohort study – PUNCH CD study - NCT01925417. Stool samples were collected prior to and	Live-JSLM dramatically reduced the abundance of antibiotic-resistant *Enterobacteriaceae* in the 2 months after administration. Fecal antibiotic resistance gene carriage decreased in

[28]	PUNCH CD		recurrent CDI who received either one or two doses of live-JSLM		at intervals up to 6 months post-therapy and analyzed by 16S rRNA gene sequencing for microbiota taxonomic composition and metagenome shotgun sequencing and bacterial culturing	direct relationship to the degree to which donor microbiota engrafted
Khanna S et al. 2022 US Overall risk of bias: Low [29]	Prospective, randomised, double-blind, placebo-controlled, phase III study PUNCH CD3 trial (NCT03244644)	44 sites in the US and Canada. Between July 2017 and February 2020	267 adults with rCDI who had completed one or more rounds of standard antibiotic therapy or had experienced two or more severe episodes of CDI resulting in hospitalisation within the past year	To demonstrate the effectiveness and safety of live-JSLM in treating patients with recurrent CDI	Patients were randomised 2:1 to receive live-JSLM or a placebo (normal saline) rectally after completing a full course of antibiotic treatment for rCDI	Live-JSLM had a higher treatment success rate than placebo (70.6% versus 57.5%) and was well tolerated with no treatment-related severe adverse events
Feuerstadt P et al. 2024 US Overall risk of bias: Some concerns [30]	Post hoc analysis included patients included in the PUNCH CD3 trial	44 sites in the US and Canada. Between July 2017 and February 2020	86 patients from the 267 adults with rCDI enrolled in the PUNCH CD3 trial	To investigate impact of live-JSLM on health-related quality of life among patients at first CDI recurrence, using data from the PUNCH CD3 trial	Health-related quality of life was measured using the *Clostridioides difficile* Quality of Life Survey (Cdiff32).<br><br>Absolute scores and change from baseline in total and domain (physical, mental, and social) scores were summarized and compared between arms. Analyses were conducted for the	Live-JSLM was associated with significantly higher Cdiff32 total (change score difference 13.5 [standard deviation 5.7], $p < 0.05$) and mental domain scores versus placebo, from baseline to week 8

					trial's blinded phase only	
Garey KW et al. 2023 US Overall risk of bias: Some concerns [31]	Secondary analysis of the randomized, double-blind, placebo-controlled phase 3 trial PUNCH CD3	44 sites in the US and Canada. Between July 2017 and February 2020	185 patients from the 267 adults with rCDI enrolled in the PUNCH CD3 trial	To investigate impact of live-JSLM on health-related quality of life among patients at first CDI recurrence, using data from the PUNCH CD3 trial	The disease-specific *Clostridioides difficile* Quality of Life Survey (Cdiff32) was administered at baseline and at weeks 1, 4, and 8. Changes in Cdiff32 total and domain (physical, mental, social) scores from baseline to week 8 were compared between live-JSLM and placebo and for responders and nonresponders	Live-JSLM -treated patients showed significantly greater improvements in mental domain than those receiving placebo. In adjusted analyses, live-JSLM -treated patients showed greater improvements than placebo in total score and physical and mental domains (all $p< 0.05$)
Feuerstadt P et al. 2023 US Moderate quality (7) [32]	Retrospective study	5 study sites, between November 2015, and September 2019	94 patients with CDI	To evaluates the safety and efficacy of live-JSLM patients with CDI	The primary end point was the number of patients with live-JSLM -related treatment-emergent adverse events	There were no serious adverse events related to live-JSLM or the administration procedure.<br>82.8% of live-JSLM -treated patients responded at 8 weeks, of whom 88.7% had sustained response through 6 months
Feuerstadt P et al. 2025 US Overall risk of bias: Some concerns [33]	Prospective, open-label, phase III trial (PUNCH CD3–OLS; NCT03931941)	US and Canada. Between July 2019 and December 2022	793 participants with a current or past diagnosis of rCDI	The primary endpoint was the number of participants with live-JSLM - or administration-related treatment-emergent adverse events.	live-JSLM was rectally administered as a single 150 mL dose 24–72 hours after completing CDI antibiotic therapy, without bowel preparation. Participants were eligible to receive a second course of single–dose live-JSLM within 21	Overall, 793 participants were enrolled, of whom 697 received live-JSLM . Adverse events through 8 weeks after administration were reported by 47.3% of participants; most events were mild or moderate gastrointestinal

				Secondary endpoints included treatment success and sustained clinical response, at 8 weeks and 6 months	calendar days if they met the criteria for treatment failure	disorders. Serious adverse events were reported by 3.9% of participants. The treatment success rate at 8 weeks was 73.8%; in participants who achieved treatment success, the sustained clinical response rate at 6 months was 91.0%
Allegretti JR et al. 2025 US Overall risk of bias: Some concerns [34]	Subgroup analysis of the PUNCH CD3-OLS (NCT03931941)	US and Canada. Between July 2019 and December 2022	74 participants with ulcerative colitis, Crohn's disease, or unspecified inflammatory bowel disease who had rCDI	To evaluate the safety and efficacy of live-JSLM in participants with rCDI and inflammatory bowel disease	Treatment-emergent adverse event data were collected for up to 6 months following live-JSLM administration	Serious adverse event within 8 weeks of administration were reported by 1.4% and 4.2% of participants with and without inflammatory bowel disease, respectively. The treatment success rate at 8 weeks was 78.9%, and the sustained clinical response rate at 6 months was 91.1%
Alonso CD et al. 2025 US Overall risk of bias: Some concerns [35]	Subgroup analysis of the PUNCH CD3-OLS (NCT03931941)	US and Canada. Between July 2019 and December 2022	141 participants who were enrolled in PUNCH CD3-OLS and were included in the immunocompromised subgroup	To evaluate the safety and efficacy of live-JSLM in participants with rCDI who were considered mildly to moderately immunocompromised	Treatment-emergent adverse event data were collected for up to 6 months following live-JSLM administration	Live-JSLM is safe and efficacious for the prevention of rCDI in participants with mildly to moderately immunocompromising conditions
Lee C et al. 2023 US	Integrated safety analysis of 5 different	US and Canada, between 30 July 2019 and	1061 participants in total	To provide cumulative safety data from five prospective	The safety population was defined as any participant who received study treatment (RBL or	Adverse events were reported in 60.2% of placebo only participants and 66.4% of live-JSLM participants.

Overall risk of bias: Some concerns [36]	trials: PUNCH CD, PUNCH CD2, PUNCH Open-Label, PUNCH CD3, PUNCH CD3-OLS	25 March 2022		clinical trials evaluating live-JSLM: 3 phase II trials (PUNCH CD, PUNCH CD2, PUNCH Open-Label) and 2 phase III trials (PUNCH CD3, PUNCH CD3-OLS)	placebo). Because participants may have received a combination of placebo and live-JSLM, participants were assigned to only one of the four treatment groups for the safety analysis	Only abdominal pain, nausea, and flatulence were significantly higher in the live-JSLM group compared with the placebo group.<br>Across five clinical trials, live-JSLM was well tolerated in adults with rCDI
Feuerstadt P et al. 2024 US Overall risk of bias: Some concerns [37]	Subgroup analysis of the PUNCH CD2 (NCT02299570) and PUNCH CD3 (NCT03244644) studies	44 sites in the US and Canada. Between 2017 and 2020	The combined PUNCH CD2 and PUNCH CD3 populations included a total of 216 live-JSLM - treated participants and 128 placebo-treated participants	To analyze clinically important risk factors that may have impacted the efficacy and safety outcomes of live-JSLM	Treatment success rates across subgroups for PUNCH CD3 were estimated using a Bayesian hierarchical model, borrowing data from PUNCH CD2.<br>Treatment-emergent adverse events were summarized for the double-blind treatment period within 8 weeks	Treatment differences between live-JSLM and placebo at 8 weeks were similar to the total population for most subgroups.<br>The largest reductions in the rate of rCDI with live-JSLM versus placebo were observed for participants with a 3-day CDI antibiotic washout period and participants with ≥4 previous CDI episodes
Knapple WL et al. 2024 US Moderate quality (7) [38]	Retrospective analysis of electronic medical records	5 study sites in the US, between 1 November 2015 and 30 September 2019	10 participants	To evaluate the safety and efficacy of live-JSLM administration via colonoscopy	The number of participants with treatment or procedure-emergent adverse events was evaluated. Treatment success and sustained clinical response were defined as the absence of CDI recurrence within	Adverse events were experienced by 75% (6/8) of participants; most were mild to moderate in severity, and none due to live-JSLM or its administration. Most participants had treatment success (80%; 8/10); 75% (6/8)

					8 weeks and 6 months, respectively	had sustained clinical response. Safety and efficacy of live-JSLM administered via colonoscopy were consistent with clinical trials
Khanna S et al. 2025 US Overall risk of bias: Some concerns [39]	Single-arm, exploratory phase III trial - CDI-SCOPE trial (NCT05831189)	12 sites in the US.	41 adults with rCDI who received one administration of live-JSLM	To assess the safety and clinical effectiveness of live-JSLM when administered via colonoscopy to adults with rCDI	Administration of live-JSLM consisted of one 150 mL dose. The primary endpoint assessed live-JSLM -related treatment-emergent adverse events within 8 weeks	5 adverse events in 4 participants (9.8%) were assessed as related to live-JSLM , all of which were gastrointestinal and mild in severity. Overall, 18 participants (43.9%) experienced 33 adverse events within 8 weeks, most of which were of mild (25/33; 75.8%) or moderate (5/33; 15.2%) severity. No adverse events led to intensive care unit admission or death
Papazyan R et al. 2023 US Overall risk of bias: Some concerns [40]	Subgroup analysis of the PUNCH CD2 study	US and Canada, 2015	27 patients with rCDI, participants in the double-blinded, placebo-controlled clinical trial PUNCH CD2	To assess the functional impact of the microbiome changes observed with live-JSLM treatment. To measure changes in fecal BA compositions from participants in the PUNCH	A liquid chromatography tandem mass spectrometry method was developed to extract and quantify 35 bile acids from 113 participant stool samples	After live-JSLM administration, there was a significant drop in primary bile acids concurrent with increased secondary bile acids that sustained through 24 months post- live-JSLM

				CD2 Phase 2 trial		
Blount KF et al. 2025 US Overall risk of bias: Some concerns [41]	Subgroup analysis of the PUNCH CD3 trial (NCT03244644)	44 sites in the US and Canada. Between 2017 and 2020	180 participants from the PUNCH CD3 trial	To assess the functional impact of the microbiome changes observed with live-JSLM treatment	Stool samples from participants in PUNCH CD3 who received a single blinded dose of rectally administered live-JSLM or placebo were sequenced to determine microbial community composition and calculate the Microbiome Health Index for postantibiotic dysbiosis. The composition of bile acids in the same samples was quantified by liquid chromatography–mass spectrometry	Clinical success after administration correlated with shifts to predominantly *Bacteroidia* and *Clostridia*, a significant increase in microbiome health index for postantibiotic dysbiosis, and a shift from primary to secondary bile acids. Several microbiota and bile acids changes were more extensive in live-JSLM -treated responders as compared with placebo-treated responders, and microbiota changes correlated with bile acids changes

CDI: *Clostridioides difficile* infection. rCDI: recurrent *Clostridioides difficile* infection.
